# Understanding super engaged users in the 10,000 Steps online physical activity program: A qualitative study

**DOI:** 10.1371/journal.pone.0274975

**Published:** 2022-10-21

**Authors:** Corneel Vandelanotte, Cindy Hooker, Anetta Van Itallie, Anum Urooj, Mitch J. Duncan

**Affiliations:** 1 Appleton Institute, Central Queensland University, Rockhampton, Queensland, Australia; 2 Priority Research Centre for Physical Activity and Nutrition, School of Education, The University of Newcastle, Callaghan, New South Wales, Australia; University of Sheffield, UNITED KINGDOM

## Abstract

**Objective:**

Sustained engagement with Internet-based behavioural interventions is crucial to achieve successful behaviour change outcomes. As this has been problematic in many interventions, a lot of research has focused on participants with little or no engagement. However, few studies have attempted to understand users with continuous long-term engagement, the so called ‘super engaged users’, and why they keep on using programs when everybody else has long stopped. Therefore, the aim of this research was to qualitatively examine characteristics, usage profile and motivations of super engaged users in the 10,000 Steps program.

**Methods:**

Twenty 10,000 Steps users (10 with more than 1 year of engagement, and 10 with more than 10 years of engagement) participated in semi-structured interviews, that were transcribed and thematically analysed.

**Results:**

Participants were aged 60 years on average, with more than half being overweight/obese and/or suffering from chronic disease despite logging high step counts (219 million steps per participant on average) on the 10,000 Steps platform. Participants indicated that the reasons for sustained use were that engaging the program had become a habit, that the program kept them motivated, and that it was easy to use. Few participants had suggestions for improvement or expressed there were program elements they did not like. Uptake of program innovations (e.g., app-version, use of advanced activity tracker instead of pedometer) was modest among the super engaged users.

**Conclusion:**

The findings from this study emphasise the need for digital health programs to incorporate features that will support the development of habits as soon as participants start to engage with the program. While a program’s usability, user-friendliness and acceptability are important to engage and retain new users, habit formation may be more important for sustained long-term engagement with the behaviour and the program.

## Introduction

In 2005 Eysenbach formulated the so called ‘law of attrition’ following the observation that in any eHealth trial a substantial proportion of users drop out before study completion, don’t use the application as intended or stop using the application prematurely [1]. This paper argued the need for a ‘science of attrition’, to develop models and theories to explain the discontinuation of eHealth application use and participants dropping out of eHealth Trials, as well as for the adoption of usage metrics to measure, analyse and discuss the determinants of attrition. Eysenbach also emphasised the need to analyse and report the characteristics of the subpopulation for which the application eventually ‘works’ (i.e., those who continue to use the eHealth application). To date, a lot of research did indeed focussed on attrition in eHealth trials [2–6], however few have examined those users with continued engagement over a long period of time and for whom the application actually works, the so called ‘super engaged users’.

Despite the many innovations in e- & mHealth that have occurred since Eysenbach’s paper in 2005 (e.g., the invention of the smartphone and associated apps) [1], high attrition and low participant engagement remains a persistent issue today [6, 7]. The importance of the problem was underscored by research demonstrating positive associations between usage of, engagement with and/or exposure to e- & mHealth applications and their effectiveness; digital interventions are more effective for those who use and engage with the application more often and over a longer period of time [6–9]. Subsequent research indicates that applications with a higher level of interactivity (for example, where users have to set goals or self-monitor activity levels), with a user-friendly design and good use of communication and persuasion practises lead to more engagement and higher effectiveness [10, 11]. Yet, despite this knowledge and the development of many innovative programs, attrition is still high in many applications [12]. Hence, the search for e- & mHealth interventions that are able to create and sustain engagement long enough to result in lasting behaviour change is ongoing.

The 10,000 Steps Australia program is no exception. The program was first developed in 2001 as a whole of community physical activity promotion intervention [13, 14], however since 2003 the implementation of the program has focussed on the use of websites, and since 2009 the program was also implemented via smartphone apps [15]. As of October 2021, the program has registered over 547,000 participants who collectively over logged 287 billion steps (www.10000steps.org.au). In its current configuration the 10,000 Steps program allows steps to be manually logged (if tracked by traditional pedometers), as well as automatically synced from advanced activity trackers (e.g., Fitbit, Garmin, Google Fit) to encourage physical activity self-monitoring and goal setting. A detailed description of the 10,000 Steps program can be found elsewhere [16]. Usage patterns of 10,000 Steps users were examined previously, and show that on average participants use the program for 45 days–which can be considered one metric of successful engagement [4]. Furthermore, 10,000 Steps users who use more interactive features (e.g., individual challenge) or advanced activity trackers (e.g., Fitbit) engage with the program for longer and log more steps [2, 17].

While the majority of registered 10,000 Steps members stop using the program after a period of time, the program also has some very loyal users, who have been with the program for many years and logged millions of steps. Usage statistics indicated that to date over 37,000 participants logged steps on more than 100 separate days, over 7,000 participants logged steps on at least 365 separate days (i.e., logging steps for at least one or more years), and over 170 participants logged steps on at least 3,650 days (i.e., logging steps for at least 10 or more years). The participant with the longest engagement has logged steps for more than 15 years. While non-usage attrition was well studied in many programs [5], including the 10,000 Steps program [4], few studies have examined super engaged users. Hence, there is a lack of understanding of why some people demonstrate very high levels of engagement when most other users have long stopped using the application. We need a better understanding of the characteristics of these users and the factors that drive their high levels of engagement to inform the creation of applications that work better for all users. Therefore, this study aimed to qualitatively examine characteristics, usage profile and motivations of super engaged users in the 10,000 Steps program.

## Materials and methods

### Participants and procedure

The database that supports the 10,000 Steps platform, and that includes information about registered 10,000 Steps members was used to select participants for this study. To be eligible participants needed to be listed in the database and have logged steps via the program website or smartphone app on a minimum of 365 separate days since tracking of steps was recorded on the 10,000 Steps website. To ensure a diversity of participants, and not only interview the most engaged super users, participants were divided into two groups: in the first group 10 participants were recruited who logged steps on more than 365 days, but less than 3650 days; in the second group 10 participants were recruited who logged steps on more than 3650 days. An attempt was made to recruit equal numbers of participants across different age groups, genders and geographical locations (i.e., urban or regional within Australia). To achieve this, the spreadsheet with data of registered 10,000 Steps participants was sorted by number of days with steps logged, gender, age, location and the date they last used the program. When there were multiple potential participants we could invite to participate, the participant who had most recently used the 10,000 Steps program was invited to participate first. If they did not respond or declined, the next person on the list was approached. E-mail was used to invite eligible 10,000 Steps members; up to 3 reminder e-mails were sent before selecting another potential participant. An appointment to conduct a telephone-based semi-structured interview was organised when potential participants responded to the e-mail invitation and agreed to participate. The e-mail also included a Participant Information Sheet to fully inform participants about the aims and nature of the study, as well as an informed consent from and a brief demographics questionnaire, that needed to be returned to the research team before the interview was conducted. Participation was completely voluntary, and participants were informed that they could withdraw anytime throughout the interview. All interviews were recorded, professionally transcribed verbatim by www.rev.com, checked for quality (CH) and then thematically analysed (AU,CV). Those who completed the study were eligible to participate in a random draw for the chance to win one Garmin activity tracker (value $180 AUD). The Central Queensland University Human Ethics Research Committee provided ethical approval to conduct this study (approval number 0000021834).

### Measures

Data obtained through the 10,000 Steps website was: date of registration (i.e., membership duration), number of step log entries, total steps logged, age, gender, and use of advanced activity trackers. Data obtained through the demographics survey was: height and weight used to calculate Body Mass Index (BMI), educational attainment, work status, health status, presence of chronic disease and the behavioural automaticity index score. The four item self-report behavioural automaticity index (SRBAI) was used to determine to what extent the physical activity behaviour of participants was automatic [18, 19]. Participants were asked to respond to the following statement: ‘Being active is something….’, 1) ‘I do automatically’, 2) ‘I do without having to consciously remember’, 3) ‘I do without thinking’ and 4) ‘I start doing before I realize I’m doing it’. Items were rated on a 7-point Likert scale, ranging from ‘1 = Strongly disagree’ to ‘7 = Strongly agree’. The scores were summed (max score = 28) and then converted to a scale where 100 represents the highest score, indicating extremely high behavioural automaticity. The SRBAI is a systematically developed automaticity scale and demonstrated content validity and reliability [19].

Prior to conducting the interviews, the researchers had developed an interview guide (see Table 1). Interview questions were designed to explore attributes associated with prolonged participant engagement with the 10,000 Steps program. The development of the open-ended questions was guided by O’Brien and Toms’ (2008) conceptual model of engagement and centered on why participants use the 10,000 Steps program [20], what their motivations are for long-term engagement with the program, how they normally use the program, what they liked and did not like about the program, and how they think the program could be improved. Leading questions about specific program attributes were avoided, and, instead, questions were designed to allow participants to voice their own views, values, and experiences. Participants were prompted to expand on their answers and give as many details as possible using standard prompting techniques (e.g., requesting more information, paraphrasing, and using affirmative noises).

**Table 1:** Interview guide.

1.	**Why are you using 10,000 Steps?** Prompts: • What is in it for you? • Why did you start using 10,000 Steps? • Do you think of yourself as a champion for walking/physical activity? • Why have you used 10,000 Steps for so long? • Why did you make 10,000 Steps a habit? • Are you still benefiting from using the 10,000 Steps website/app? • How has your use of the 10k site impacted further into your life–Workplace/Community Tournament?
2.	**What do you like about 10,000 Steps?** Prompts: • What do you like most about 10,000 Steps? • Does the 10,000 Steps website/app help you to be active and stay motivated?
3.	**What do you not like about 10,000 Steps?** Prompt: • What do you really not like about 10,000 Steps?
4.	**How have you changed your use of 10,000 Steps over time?** Prompts: • Do you use trackers? • Do you use the app? • Only website–phone or computer?
5.	**How have you liked or not like the changes to 10,000 Steps over time?** Prompts: • Website redevelopments • App changes • Tracker integration
6.	**Why have you continued to use 10,000 Steps for so long?** Prompts: • Do you feel you still need 10,000 Steps to be active? • What keeps you coming back (programs, support, features, colours, OCD, habit, personality trait)? • Do you use 10,000 Steps to keep you motivated? • Would you say it is perhaps something about you or your personality that has made you use 10,000 Steps for so long, compared to other people? • Do you have a specific pattern/routine in which you use the 10,000 Steps website/app? • How do you track your steps on holidays? • Annual workplace/Community Tournament
7.	**What features of 10,000 Steps do you use?** Prompts: • Challenges, Tournaments, Buddies, Trackers, App, articles, Health Challenge • Are there features that you don’t like?
8.	**How could we make 10,000 Steps better for you?** Prompts: • Different tracker, news feed, better app, website feature?
9.	**Do you encourage others to use 10,000 Steps?** Prompts: • Have you recruited others to the website? • Do you tell other people that you use 10,000 Steps? • What do those people say? • What impact did that have on others around you?
10.	**Do you use any other trackers/apps/websites to track your physical activity/healthy eating/sleep/menstrual progress?** Prompt: • Do you use all of the different tracking sites at once or fluctuate between them?

### Analyses

Data collection was conducted from February 2020 to September 2020. Data analyses were conducted between January and February 2021. The interview recordings were analysed thematically [21, 22]. Initially, transcripts were read by one of the authors (AU) to become familiar with the data, before being read a second time as part of the coding process. After open-coding 10 transcripts, the same author (AU) developed a list of preliminary codes and definitions for each code that taken together formed a codebook. These preliminary codes were cross-checked by a second author (CV) who also read all transcripts. Findings were discussed and noted until consensus was reached. Throughout the data analysis, codes, categories and themes were continually refined in consultation with other study authors. Some codes were combined, others were split into subcategories, and new codes created until the final codes were reached. The final codes were examined and organised into a hierarchical structure. The researchers adopted a reflexive approach throughout the data collection process and analysis, by taking notes about participants’ comments and researcher’s thoughts during the interviews, memoing after the end of the interviews and through continuously exploring their own positionality in relation to the development of new knowledge [22].

The original intent was to analyse participants who logged less than 3650 days separately from those who logged more than 3650 days. However, as there was no clear distinction in how they responded to the questions in the interviews, they were all analysed as one group. Other studies using semi-structured interviews indicated to have achieved data adequacy with less than 20 participants [23–26]. Therefore, it was intended to recruit 20 participants (10 in each group) and then recruit additional participants until data adequacy was achieved. However, the researcher (CH) who conducted the interviews indicated that data adequacy was achieved before the 20 original interviews were completed; and combining participants into one group further limited the need to recruit more participants. Descriptive statistics were used to describe the sample, and Nvivo 12 was used to thematically analyse the data.

## Results

In order to recruit 20 participants, a total of 52 people were invited to participate. An equal number of men and women participated, equally distributed between those who logged steps on the 10,000 Steps platforms more or less than 3650 times (see Table 2). The average age of participants was 60 years (range: 38 to 74; 5 participants were over 70 years). The majority of participants had a higher education degree (n = 16) and were employed at the time of the study (n = 13); all those without a job were retired. The majority of participants (n = 13) had a BMI over 25 and indicated they had a chronic disease (n = 12) at the time of the study. Less than half of participants (n = 9) rated their health as ‘excellent’ or ‘very good’. Those who logged less than 3650 times had been a member for approximately 4 years on average and logged more than 32 million steps over 1275 step log entries in total. Those who logged more than 3650 times had been a member for 14 years on average and logged more than 400 million steps over 5045 step log entries in total. Most participants used a pedometer to log steps (n = 14). Only one participant with more than 3650 step log entries used an advance activity tracker to sync steps, while 5 participants with less than 3650 step log entries had done so. The SRBAI score was high in all participants (76.8), though higher among participants with more than 3650 step log entries (81.5). Five participants had the highest possible SRBAI score (100).

**Table 2:** Participant characteristics.

	Total group (N = 20)	Logged steps between 365 and 3650 times (n = 10)	Logged steps over 3650 times (n = 10)
Female (n)	10	5	5
Age (mean)	60.2	57.7	62.8
Has higher education degree (n)	16	9	7
Currently employed (n)	13	8	5
BMI (mean)	29.4	29.6	29
Has a chronic disease (n)	12	6	6
Has ‘excellent’ or ‘very good’ health (n)	9	4	5
Number of years as a member of 10,000 Steps (mean)	8.9	4.4	14.0
Number of days with steps logged (mean)	3160	1275	5045
Total steps logged (mean)	219,014,000	32,064,000	405,964,000
Have synced steps using an activity tracker (n)	6	5	1
SRBAI (%)	76.8	72.2	81.5
Maximum score (100) on the SRBAI (n)	5	2	3

Note: SRBAI = Self-Report Behavioural Automaticity Index

The 20 interviews ranged in time from 6 to 33 minutes, with the average time being 16 minutes. Nine main themes were identified: 1) reasons for starting to use the 10,000 Steps program, 2) reasons for long-term use of the 10,000 Steps program, 3) what participants liked about the 10,000 Steps program, 4) how the 10,000 Steps program can be improved, 5) what participants did not like about the 10,000 Steps program, 6) encouraging others to use the 10,000 Steps program, 7) how participants changed their use of the 10,000 Steps program over time, 8) using the 10,000 Steps program while on holiday, and 9) tracking behaviours other than physical activity.

### Reasons for starting to use the 10,000 Steps program

The majority (n = 12) of super engaged users started using the 10,000 Steps program because their workplace organised a ‘Tournament’ (a.k.a. Workplace Challenge), or because their workplace provided them with a pedometer (n = 3). A small number of people found out about the program through word of mouth (n = 3) and others started using the program because they were diagnosed with diabetes (n = 1), because they were able to use a pedometer through a library loan scheme (n = 1) or because they bought a Fitbit activity tracker (n = 1).

*“Well*, *I guess*, *it all started a number of years ago*, *it’s been quite a while*. *I couldn’t tell you how long now*. *My HR department gave out pedometers and links to the 10*,*000 Steps website*. *And that was where it all began*.*” (Participant 003*, *male*, *aged 48*, *4181 step log entries*)

*“I don’t remember*. *Years ago*, *way back in about 2004 or something like that*. *I don’t know*. *I was walking a lot*, *and I think someone said*, *"Do this 10*,*000 Steps program where you can put all your steps online*.*" And that’s how I looked it up and joined*.*” (Participant 004*, *male*, *aged 72*, *5809 step log entries*)

### Reasons for long-term use of the 10,000 Steps program

The two main reasons why the super engaged users keep using the 10,000 Steps program, is because over time using the program became a habit (n = 13) and because the program keeps them motivated to stay active (n = 13). Others indicate they keep using the program because they like how data driven it is (n = 6) or because it keeps them accountable to be active (n = 4). A couple of participants indicated they keep using the program because it helps them to keep their weight under control (n = 2), because they like the monthly Challenges (n = 1), because the program allows them to sync their steps with different devices (n = 1), because it is simple to use (n = 1) and because they are loyal to the program (n = 1).

*“Yeah*, *it’s got to be a bit of a habit*. *Almost an obsession now that I make sure I get my steps in*. *I’ve been doing it for so long that it’s part of me now*, *you know*?*” (Participant 004*, *male*, *aged 72*, *5809 step log entries*)

*“If I don’t have my pedometer*, *the physical one*, *I get a bit*, *where is it*, *I need it*. *I don’t feel like I’m complete without it*. *So really quite a habit*. *Yes*.*” (Participant 019*, *male*, *aged 47*, *2490 step log entries*)

*“So yeah*. *It’s just something that just keeps you*, *I suppose*, *motivated to keep logging the numbers I suppose*.*” (Participant 011*, *male*, *aged 54*, *4153 step log entries*)

### What participants liked about the 10,000 Steps program

When asked what the super engaged users liked about the program the most common response (n = 7) related to the simplicity of the program and its ease of use. Another common response (n = 5) was that it records all activities and provides lifetime statistics even for long-term users. Several participants (n = 4) indicated that they liked that the program motivates them to stay active. Other less common (n = 1) responses were: that it is an Australian program, that it allows to add other types of activity (e.g., minutes of cycling) that can be converted into steps, that it is designed for average people, that their cardiologist recommends they use the program, that they like the 10,000 Steps concept and the monthly Challenges, that the program allows to set daily aspirational goals, and that the program adopts new technology as it becomes available.

*“What I do like about is the app and the website are both easy to use and navigate*. *I find that useful*.” *(Participant 005*, *female*, *aged 48*, *733 step log entries*)

*“Oh*, *it gives you a goal to aspire to*, *for each day*, *and then*, *you can make your own targets*.*” (Participant 017*, *female*, *aged 56*, *5284 step log entries*)

*“I think what is good about it is like it’s kept up-to-date with the changes in technology*, *like it’s not the same as it was in 2004 or 2010*.*” (Participant 006*, *female*, *aged 66*, *5809 step log entries*)

### How the 10,000 Steps program can be improved

When asked if anything could be improved to the 10,000 Steps program, half of the participants indicated no improvements were needed. Those that did have ideas mostly suggested improvements to the monthly Challenges (i.e., add interactive maps, incorporate walking destinations known to me, offer challenges that are easier for beginners) and the 10,000 Steps smartphone app (i.e., improve usability and functionality; make individual challenges available in the app; have the app sync with more activity trackers). Some participants also had suggestions to improve the overall engagement with the platform (i.e., increase accountability towards meeting goals; improve visualisation of step log data; offer more encouragement, awards and congratulations for achievements; add more fun facts and stats about walking achievements; offer financial incentives for distance walked).

*“No*, *nothing that I can think of*. *I’m quite happy with it*. *I just put my steps on just yesterday and I can’t think of anything else that could improve*.” *(Participant 006*, *female*, *aged 66*, *5809 step log entries*)

### What participants did not like about the 10,000 Steps program

Nearly all of the Super Engaged Users (n = 16) indicated there wasn’t anything they specifically disliked about the 10,000 Steps platform. However, when those that did mention something they didn’t like, it always referred to very specific technical aspects. For example, not being able to sync trackers directly with the platform, or not liking how the leader board or step challenges are organised.

*“No*, *I can’t think of anything that is occurring that I dislike*. *I think it’s amazing how it integrates with the Fitbit effortlessly*.*” (Participant 012*, *male*, *aged 50*, *719 step log entries*)

*“I guess I could say I don’t like the fact that the Apple integration isn’t there*. *But you’ve mentioned that that’s something that might coming down the pipeline in the future*.*” (Participant 005*, *female*, *aged 48*, *733 step log entries*)

#### Encouraging others to use the 10,000 Steps program

The majority of Super Engaged Users (n = 14) did indicate that they encouraged others to walk, though the remaining participants said they didn’t do this. Some indicated that they specifically encouraged others to use the 10,000 Steps program (N = 7), whereas others encouraged the habit of walking more broadly, without specifically referring to the 10,000 Steps program (n = 2). Some indicated they had tried to get others into walking, but that it didn’t work (n = 4). Several specifically referred to encouraging their partner to walk more (n = 3).

*“Well*, *I tried*. *Tried to get people to join the team at work*, *but I don’t know*. *I think I was too much of a hard-ass*.*” (Participant 013*, *male*, *aged 38*, *777 step log entries*)

*“I do encourage other people to walk*. *But I’m not sort of preaching it*, *but it’s probably actually more neighbours and stuff like that know I walk everywhere*. *And they always say something about it and they always say to me*, *"I need to get into” They don’t always do that but sometimes they do*. *Yeah*.*” (Participant 002*, *male*, *aged 69*, *5902 step log entries*)

### How participants changed their use of the 10,000 Steps program over time

The 10,000 Steps program launched an entirely new website (different design and layout, though with similar functionality) in 2017. Nearly all participants either didn’t notice/remember the change to the latest version of the 10,000 Steps website, or they liked it better. Though one participant mentioned that she struggled at first to get used to the latest website, but got used to it quickly thereafter.

*“Yeah*, *yeah*. *No*, *I have liked the changes*. *I do it all on my iPad*, *on the website*, *I don’t use a computer or laptop*, *but no*, *I have liked the changes*. *To me the changes have kept up-to-date*.*” (Participant 006*, *female*, *aged 66*, *5809 step log entries*)

*“I found them a problem initially*, *but once I worked out*… *I can remember contacting someone once and saying*, *"how do I do something or other*?*" But once I worked it all out I was right*. *Because I’m not very technical anyway*.*” (Participant 007*, *female*, *aged 74*, *6254 step log entries*)

The 10,000 Steps program first launched a smartphone app in 2009. The usage of the app among super engaged users is mixed, with the majority indicating they don’t use it (n = 11), a couple who use both app and website (n = 4), and only a few who say they only use the app (n = 3). Of those that use the app, satisfaction is mixed, with some really liking it and others indicating the functionality of the app is too limited compared to the website.

*“No*, *you see I’m one of these prehistoric people that doesn’t own a mobile phone*.*” (Participant 008*, *female*, *aged 57*, *661 step log entries*)

*“I actually use both*. *I use the website when I’m at work*, *and I use the app when I’m at home*.*” (Participant 010*, *female*, *aged 50*, *4077 step log entries*)

*“Yeah*, *the website because I found that the*… *There’s the app and for some reason the app and the website don’t sync*. *If you put into the app it doesn’t sync to the website*. *But this was a while ago*.*” (Participant 013*, *male*, *aged 38*, *777 step log entries*)

The 10,000 Steps program first integrated the use of activity trackers including Fitbit (2017), Garmin (2019) and Google Fit (2021), to enable automatic syncing of steps, in addition to the option of manually updating step counts for those that are using a traditional pedometer or other step tracking device. While many of the super engaged users are still using pedometers (n = 8), a significant proportion are now using activity trackers (n = 9). The majority of these trackers are compatible with the 10,000 Steps website (n = 6), but some are also using trackers that don’t automatically sync (e.g., Apple Watch), meaning they still have to enter their steps to the 10,000 Steps platform manually. Several (n = 3) also indicated that they use the step counting feature of their smartphone as a backup for when their pedometer or tracker is not available.

*“I have used a variety of things*. *I’m currently using an Apple Watch*.*” (Participant 005*, *female*, *aged 48*, *733 step log entries*)

*“I find a pedometer is actually a little bit more accurate and I’m not really interested in how many calories I’m burning and stuff like that*. *But I thought the pedometer is much more accurate than the other devices*, *for some reason*. *My backup is my phone*. *It just works off GPS*, *whatever algorithm they’re using is probably reasonably good*, *but the pedometer is my go-to*.*” (Participant 002*, *male*, *aged 69*, *5902 step log entries*)

### Using the 10,000 Steps program while on holiday

Most participants (n = 11) reported that they tried to keep track of their steps during holidays. Some (n = 3) indicated it is easier to be active during holidays, as there is more time for it. Several (n = 5) participants indicated that the 10,000 Steps platform (website + app) makes it easy for them to keep recording steps, though many others (n = 7) indicated that they record their steps offline (i.e., paper-and-pencil) for a while (i.e., couple of days to up to a week) before logging them on the website. One participant indicated that they always wear their Fitbit, but only sync their steps when they take their tablet with them during the holiday.

*“But on holidays when I’ve got a lot more time*, *I go out of my way to do it*.*” (Participant 003*, *male*, *aged 48*, *4181 step log entries*)

*“Oh*, *the same*. *We are very conscious of when we go out for a walk*, *and all of our holidays are planned around walking*. *Walking is our life*, *in a way*. *We love it*. *And so we always*, *and my partner is much better at this than I am*.*” (Participant 018*, *female*, *aged 71*, *1703 step log entries*)

### Tracking behaviours other than physical activity

Many participants (n = 7) indicated that they are not tracking any other behaviours (e.g., Sleep) other than their steps and physical activity. Some (n = 5) do use other apps to track their activities (e.g., Strava, Myzon, Mapometer, Medibank Life, Drivers for Cycling).

*“No*. *I’ve thought about the sleeping part and I’ve thought*, *I’m not sure it’ll tell me what I really want to know*. *I’m not sure how it works it out to be honest*.*” (Participant 002*, *male*, *aged 69*, *5902 step log entries*)

*“Oh*, *actually I think it is an app now*, *but Mapometer*. *If you’ve ever heard of that*. *That actually measures a route so you can draw the route you take*. *So if you cut through a park*, *you can draw through that park and find out how many kilometers that particular route was*.*” (Participant 013*, *male*, *aged 38*, *777 step log entries*)

## Discussion

This study aimed to gain a deeper insight into what drives super engaged users of the 10,000 Steps program to continue to use the program, well beyond the levels of engagement typically observed in this program and many other interventions. Both participants’ scores on the SRBAI, as well as participants’ responses in relation to why they keep using the program point at the development of deeply ingrained routines and habits. Habits are automatic and impulsive processes, activated by contextual or environmental prompts, which require minimal cognitive effort, awareness, control or intention [27]. Habit strength will predict the likelihood of engaging in habitual behaviour, which was previously also demonstrated for physical activity behaviour [27]. Many participants noted the importance of habit for logging steps as a driver to keep them engaged.

As many of the super engaged users indicated that no further improvements to the program were needed, and that there was nothing they didn’t like about the program–despite significant changes to the program over time [16], it is likely that the habit formation in the 10,000 Steps super engaged users was more important than aspects of the program that are important for new users (i.e., usability, user-friendliness, acceptability) [27]. However, for new users to establish habitual self-monitoring of behaviour it is nevertheless important that the program is highly acceptable and user friendly [28], and incorporates important behaviour change techniques that demonstrate their effectiveness to achieve sustained behaviour change (i.e., self-monitoring, goal setting, social support, gamification) [29]. As such, participants also emphasised the ease of use as one of the main reasons why they liked the program, as well as self-monitoring features that capture lifelong cumulative step log data. This finding also emphasises the importance of ensuring continuity as the program goes through cycles of renewal; while new features were introduced the super engaged users were always able to use the program in the same way as when they first signed up, and their cumulative step log data was never interrupted.

Other factors that point to the importance of habit formation in the super engaged users are the low usage of the 10,000 Steps smartphone app and the high usage of pedometers (especially in those with more than 3650 step log entries, who also reported higher habit strength). This may be due to the program not having a smartphone app available prior to 2009, nor the option to log steps with advanced activity trackers prior to 2017, when they first started to use the program. It may be that these participants got used to the program without having these features and possibly didn’t feel a need to change how they interact with the program. However, it is also important to note that several participants did mention the limited functionality of the app (e.g., no ability to engage in monthly Challenges which was only added recently) that may have prevented adoption of this feature. Despite the lower adoption of the 10,000 Steps app in super engaged users, our previous research nevertheless demonstrated the importance of the app in improving engagement with the program and offering users a range of options on how they use the program.^4,17^ Likewise, there are distinct benefits to using traditional mechanical pedometers over more advanced activity trackers. Pedometers are significantly simpler in use: typically one function only (i.e., tracking steps) compared to a plethora of functions (e.g., sleep tracking, heart rate tracking, GPS tracking) [30]. Good quality pedometers (e.g., Yamax Digi walker SW200, which is recommended for use by the 10,000 Steps program) demonstrated high accuracy in tracking steps, comparable to more advanced activity trackers [31]. Pedometers are robust, have a long life and don’t need to be recharged on a daily or weekly basis (i.e., a single battery can last several years) [32]. While pedometers don’t automatically sync their steps to the 10,000 Steps platform, users don’t have to worry about installing apps, syncing steps and Bluetooth connectivity. As such, there are plenty of reasons why super engaged users may simply prefer to continue using pedometers, especially older users that may be less tech-savvy [33].

To a large extent the success of the 10,000 Steps program was driven by the very large adoption of the program by workplaces (over 18,000 workplaces to date) through their participation in ‘Tournaments’ (a.k.a. ‘workplace challenges’, where teams challenge each other to take as much steps as possible over a set period) [16]. The importance of Tournaments was also demonstrated for the super engaged users, as for many it was the primary reason why they initially started to use the 10,000 Steps program. Unlike the super engaged users, many 10,000 Steps members stop using the program when a Tournament is completed, well before habit formation is established. This is unfortunate and efforts need to be made to retain more participants after the completion of Tournaments, so that they can also benefit from the long-term benefits of physical activity participation [34]. As such, the suggestions made by the super engaged users with regards to enhancing the implementation of the challenges on the 10,000 Steps platform should be closely examined. Several of those suggestions encouraged the development of Challenges with more interactive features. This is in line with our previous research that suggests that features on the 10,000 Steps website that incorporate more interactive elements are associated with higher engagement levels [2].

Finally, while some of the super engaged users indicated that they specifically started using the program in order to manage a chronic disease, the high level of overweight and obesity among the participants, as well as the high prevalence of chronic disease among participants was a surprising observation. Given the sustained high engagement with this physical activity program (i.e., participants logging many millions of steps logged on the platform), and given the demonstrated effectiveness of the program [13, 14], a lower level of overweight/obesity and chronic disease was instinctively expected. However, the average age of participants in this study was high (60 years on average), and chronic disease is very common in older people. Though perhaps it also illustrates that, while physical activity is very important to reduce the impact of chronic disease and significantly improve quality of life [34], adherence to physical activity alone is not sufficient to prevent chronic disease. It is also important to pay attention to other health behaviours (i.e., diet, sleep, smoking, alcohol consumption) [35], and it may be that study participants focussed predominantly on logging steps and ignored adhering to other important health behaviours. As such, this observation aligns with often used sayings ‘you cannot outrun your diet’ (or ‘outwalk’ it) and ‘it is better to be fat but fit, than to be thin but unfit’ [36]. As we did not expect so many of the participants reporting a chronic disease or overweight, we did not ask any questions about health behaviours other than physical activity. However, future research should aim to gain a broader view of participants’ health behaviours.

A strength of this study relates to the inclusion of participants with extremely high levels of engagement with a health promotion program over a long time period. The experiences of super engaged users are rarely reported, often due to the lack of access to such users given high non-usage attrition reported in many digital health programs [12], as well as the lack of health promotion programs that have operated over a timespan as long as that of the 10,000 Steps program (i.e., 20 years). Further strengths relate to the robust approach to thematically analysing and reporting the data, as well as the use of a reliable and valid tool to report on habit strength (i.e., the SRBAI). However, due to the qualitative nature of this study, it is not possible to generalise the findings more broadly. Although thematic saturation was achieved with fewer than 20 participants, it is possible the views reported by the participants are not representative of all super engaged users for the 10,000 Steps program due to the sampling method applied. However, the average age of all super engaged users aligned well with those included in the study (i.e., average age for all super engaged users with more than 365 step log entries is 58 years; average age for those with more than 3650 step log entries is 66 years). As we strived for equal male and female participant numbers (i.e., ten of each), the gender distribution of all super engaged users with more than 365 step log entries (54% female) and those with more than 3650 step log entries (41% female) was less aligned to those included in the study. Furthermore, the high average age of the super engaged users may have reduced their adoption of new innovations in the 10,000 Steps program (i.e., app use, tracker use). Additionally, knowledge about participants’ other health behaviours (e.g., diet, smoking) would improve the interpretation of our findings, especially in relation to chronic disease. Finally, while 10,000 Steps members are encouraged to use the program as long as possible, to help with habit formation, it should be pointed that the program aims to transfer new skills and knowledge to its participants so they can learn how to be physically active without the need (or dependency) of the program in the long term.

In conclusion, the super engaged users demonstrated very high levels of habit strength. This emphasises the need for digital health programs to incorporate features that will support the development of habits as soon as participants start to engage with the program. Over time, the power of habit appears to become more important than a program’s usability, user-friendliness and acceptability or latest new features. However, as all users are new users at some point in time, the importance of having a highly convenient and user-friendly program should not be underestimated or ignored. When a program goes through cycles of renewal it is equally important that it doesn’t disrupt the habits of its super engaged users, so they can keep using the program as they always have.

## Supporting information

S1 Data (CSV)
